# The Mediterranean Diet in Osteoporosis Prevention: An Insight in a Peri- and Post-Menopausal Population

**DOI:** 10.3390/nu13020531

**Published:** 2021-02-06

**Authors:** Sara Quattrini, Barbara Pampaloni, Giorgio Gronchi, Francesca Giusti, Maria Luisa Brandi

**Affiliations:** 1 Experimental and Clinical Biomedical Sciences “Mario Serio”, University of Florence, Viale Pieraccini 6, 50139 Florence, Italy; sara.quattrini@unifi.it (S.Q.); barbara.pampaloni@unifi.it (B.P.); francesca.giusti@unifi.it (F.G.); 2 Department of Neurosciences, Psychology, Drug Research, and Child Health (Section of Psychology), University of Florence, Via di San Salvi 12, 50139 Florence, Italy; giorgio.gronchi@unifi.it

**Keywords:** osteoporosis, Mediterranean diet, calcium intake, menopause, bone health

## Abstract

Osteoporosis represent a widespread public health problem. The management and prevention of osteoporosis and related low energy fractures start with a correct lifestyle and proper nutrition. Several different nutrients are essential for bone and mineral metabolism, especially calcium. Nevertheless, a well-balanced nutrition, such as Mediterranean diet (MD), proved to be beneficial for several chronic diseases and also fragility fractures resulted lower in the Mediterranean area. A prospective observational study in a population of two hundred peri- and post-menopausal women (aged 30–80 years) was developed at Careggi hospital, Florence. Both MD adherence and dietary calcium intake were evaluated in occasion of a “first visit” and a “follow-up” visit, through validated questionnaires. From a descriptive point of view, although not statistically significant, in both visits a slight increase in calcium intake was observed for high adherence to MD diet. Moreover, a short nutritional interview (20 min) was applied in our population and demonstrated to be sufficient to significantly improve MD adherence level (mean score at T_0_ = 6.98 ± 1.74 and T_1_ = 7.53 ± 1.68), opening promising paths in osteoporosis prevention.

## 1. Introduction

Osteoporosis (OP) and fragility fractures represent a public health problem in western countries, involving a large part of the population and steadily increasing worldwide. In fact, OP is a chronic condition that can require long-term management, and the World Health Organization (WHO) describes it as a “progressive systemic skeletal disease characterized by low bone mass and microarchitectural deterioration of bone tissue, with a consequent increase in bone fragility and susceptibility to fracture” [1].

Globally, OP affects over 200 million individuals. Hernlund E. et al. reported in 2010 that, in the European Union, approximately 6% of men and 21% of women aged 50–84 years have osteoporosis, affecting 27.6 million men and women [2].

In Italy, it is estimated that there are about 3.5 million women and 1 million men suffering from OP. Given that the percentage of the Italian population over 65 years of age will increase by 25% over the next 20 years, we can expect a proportional increase in the incidence of osteoporosis [3]. 

Low-energy fractures, typical of osteoporosis, represent the main clinical evidence of disease progression. Major fragility fractures (especially hip fracture) increase mortality risk in all ages, and in older age also minor fractures increase the risk [4]. 

Tarantino et al., in their study based on a three-year multicenter survey, estimated that in Italy there is an annual incidence of 410,000 new hip, humeral, wrist, ankle, and vertebral fragility fractures. The authors observed that about 70% of the overall fractures observed during the study period occurred in persons aged older than 65 years, and 87% were classified as fragility fractures [5].

Management and prevention of osteoporosis and related low energy fractures starts with a correct lifestyle, that includes smoking abstention, daily physical activity, low alcohol consumption and proper nutrition. In this manuscript, we will analyze the relationship between Mediterranean diet, calcium intake and bone health. The results from a prospective cohort study, considering a population of peri- and post-menopausal women, will be reported, in order to clarify the relationship between diet and bone health, with the aim to examine Mediterranean diet adherence level and dietary calcium intake.

### Background

Diet is of critical interest in osteoporosis because it is one of the few safely modifiable risk factors. Healthy, well-balanced nutrition can play an important role in the prevention and pathogenesis of osteoporosis, and in support of pharmacological therapy [6]. Several factors contribute to the development of osteoporosis in post-menopausal women, among which, low-grade inflammation, poor dietary habits, and sedentary and unhealthy lifestyles, such as smoking and alcohol consumption [7,8].

Calcium is the main actor in bone health. It is the principal component of the mineralized bone matrix where more than 99% of the total body calcium is contained; its key role in maintaining bone health throughout life has been recognized by many studies. An optimal dietary calcium intake is necessary for bone health at all stages of life: In children and adolescents to contribute to the formation of a healthy skeleton, and in adults and the older adults to allow the maintenance of adequate bone mass [9,10]. Dietary requirements for calcium are determined by the need for bone development and maintenance; therefore, Recommended Dietary Allowance for Calcium varies throughout life. As stated both at the international level by the Institute of Medicine of the National Academy of Sciences (IOM) [11], and at the Italian level by the Reference Levels of Nutrients and energy intake for the Italian population (LARN, Livelli di Assunzione di Riferimento di Nutrienti ed energia per la popolazione italiana) (Tb.1) [12], the recommended daily requirements of calcium are 1000 mg for adults (male and female) and 1200 mg for subjects older than 65 years, teens, and those who suffer from osteoporosis.

Nutritional intake is the preferred method for calcium acquisition, because it allows the introduction of small quantities of the mineral throughout the day. This way, its absorption is optimized, avoiding oscillator spikes that could lead to cardiovascular complications [13].

Although dietary calcium intake is essential for skeletal health, a recent review by the International Osteoporosis Foundation (IOF) showed that, in 74 countries around the world, average calcium intake ranges between 175 and 1233 mg/day, with values sometimes much lower than those recommended for the adult population. Only in northern Europe do the average values exceed 1000 mg per day [14]. In Italy, the 2005–2006 INRAN-SCAI study on women and men aged from 18 to 65 years reported that the average calcium intake in the male population was 799 ± 337 mg/day, while for the female population it was 730 ± 277 mg/day, both below the LARN recommendations for adults [15].

Over the last 20 years, numerous epidemiologic and experimental studies in nutrition have focused on the Mediterranean Diet (MD), because it is well-known for its health benefits and protection against several chronic western diseases (cardiovascular and metabolic diseases) [16,17].

The incidence of OP and fragility fractures is very variable in the countries of the European Union, but it has been observed that it is lower in the Mediterranean area [18]. The CHANCES project [19], the EPIC [18] and the EPIC-older study [20], the Women’s Health Initiative [21], and some Swedish population studies [22,23], report that the incidence of fragility fractures is lower in subjects with a diet more adherent to the Mediterranean pattern.

However, a direct cause-and-effect association between adherence to the MD and a reduction in fragility fractures incidence has not been demonstrated.

Recently, scientific literature reviews have confirmed that greater adherence to the MD is associated with a reduced total fracture risk and higher bone mineral density (BMD) [24]. However, the beneficial effect of the Mediterranean diet on skeletal health is more controversial when the observational studies are analyzed as a whole. A review by Kunustor et al. reports that there are still limited observational studies that support beneficial effects of adherence to the Mediterranean lifestyle on the incidence of hip fractures [25].

Despite the conflicting results, interest in studying the impact of nutrition and, in particular, of MD on bone health, is still very high. A recent longitudinal cohort study by Benetou et al. examined 140,775 adult subjects from five cohorts (Europe and the United States) to assess the level of adherence to the MD and the incidence of fractures. From this population, it emerged that the incidence of hip fracture was lower in those with medium and high levels of adherence to MD than in those with low adherence [26].

The traditional Mediterranean diet is characterized by a high intake of vegetables, legumes, fruits and nuts, cereals (which, in the past, were largely unrefined), a high intake of olive oil (and a low intake of saturated lipids), a moderately high intake of fish (depending on the proximity to the sea), a low-to-moderate intake of dairy products (mostly in the form of cheese or yogurt), a low intake of meat and poultry, and a regular but moderate intake of ethanol, primarily in the form of wine during meals.

Current research shows that the consumption of food groups typical of the Mediterranean diet, such as fruit, vegetables, low-fat dairy products, and fish, is essential for maintaining good bone health [27,28,29,30].

The use of extra virgin olive oil (EVOO) as the main source of fat has been shown to be beneficial in preventing bone loss, probably due to the high content of polyphenols. An in vitro study by García-Martínez et al. shows that EVOO phenols can modulate the cellular proliferation and maturation of osteoblasts, increasing the activity of alkaline phosphatase and depositing calcium ions in the extracellular matrix [31].

However, few studies have been performed on humans. Fernández-Real et al. evaluated the effects of olive oil consumption on circulating levels of osteocalcin (OC). An MD enriched with EVO, administered to the subjects for two years, was associated with a significant increase in blood OC and concentrations of the N-terminal pro-peptide of type 1 procollagen, suggesting a protective effect on the bone [32].

The results in an additional study, although preliminary [33], show a well-defined image of the health properties of MD on prevention and slowing progression of chronic degenerative diseases, including OP.

## 2. Materials and Methods

Starting from this background knowledge, our group performed a study to clarify the relationship between diet and bone health, specifically in a female population characterized by several variables. In particular, the aim of the study was to examine Mediterranean Diet (MD) adherence level and dietary calcium intake in peri- and post-menopausal women who visited the Bone and Mineral Metabolism Unit in Careggi Hospital, Florence, in order to investigate the role of the MD for bone health maintenance.

### 2.1. Subjects and Procedures

A prospective, observational, monocentric, spontaneous, no-profit study was performed in Careggi Hospital, Florence, Italy, from September 2017 through August 2018. The study was approved by the Institutional Review Board (Comitato Etico Area Vasta Centro, Azienda Ospedaliera Universitaria Careggi, Florence, Italy) [number: 11097_oss]. The Ethics Committee verified the conformity to the *Good Clinical Practices* and to the Declaration of Helsinki. All patients gave informed consent for participation in the study and consent for data publication.

**Figure 1:**
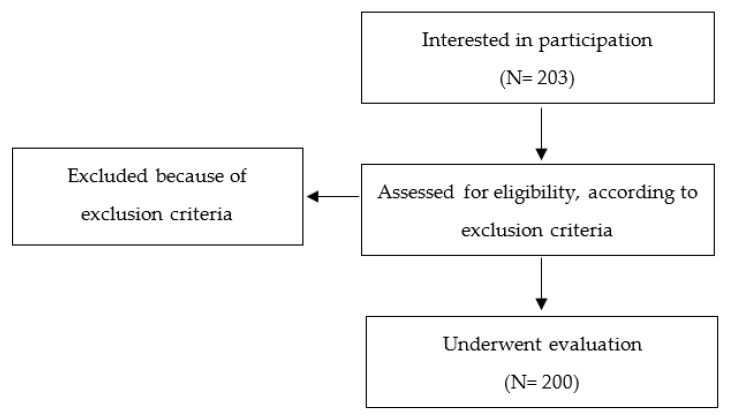
Flow chart showing a description of study design, recruitment and assessment.

Two hundred peri- and post-menopausal women, age range 30–80 years, were recruited upon their “First visit” to the Bone and Mineral Metabolism Unit (Careggi Hospital, Florence). The exclusion criteria included: Pregnancy and breastfeeding, and participation in other studies. Figure 1 shows the recruitment procedures. Recruited subjects were evaluated a second time in occasion of a “Follow-up visit”. The time distance between “first visit” and “follow-up visit” was variable from one to twelve months and was decided by the specialist on the basis of the “first visit” results and each subject’s needs.

In occasion of the “First visit” to the Unit, a trained nutritionist presented the study and gave the documents necessary for participation (patient information pack, informed consent, and consent for data handling), which were then signed by the participants. Information regarding socio-demographic data, clinical assessment (pathologies, medical treatments or supplement use), lifestyle habits (physical activity index, smoking habit), were then recorded.

The nutritional investigation was performed through two specific questionnaires. First, a 14-item questionnaire was administered to evaluate MD adherence level, examining the consumption of typical Mediterranean food, such as olive oil, fruits and vegetables, and legumes [34]. One point was assigned to each correct Mediterranean diet behavior, and the total score ranged from 0 to 14: A total score equal to or under five corresponded to low MD adherence, between 6 and 9 medium MD adherence, and equal to or above 10 high MD adherence.

Second, a semi-quantitative Food Frequency Questionnaire (FFQ) was administered to evaluate calcium intake. The questionnaire consisted of 15 food items, including: milk and dairy products, fruits, vegetables, legumes, cereals, meats, fish, eggs, and calcium-rich mineral water [35]. Subjects were asked to report consumption frequency and portion size assessment. Calcium intake was assessed by a specific worksheet, using Microsoft Excel 2010 software, which reported the calcium content of each food, referring to Food composition Tables of the Centre of Research for Food and Nutrition [36] and Food Composition Database for Epidemiological Studies in Italy of the European Institute of Oncology [37].

A trained nutritionist performed the evaluation and data collection procedures. After that, the nutritionist gave dietary advices of *good clinical practice* to the participants. In particular, a short conversation (15–20 min) was dedicated to advice for good dietary habits for bone health, focusing on calcium intake and improving MD adherence.

During the “Follow-up visit” at the Unit, information regarding medical treatment and/or supplements, blood sample results, instrumental exams, and lifestyle habits were collected again. The nutritional evaluation was also repeated. Anthropometric measurements (weight, height, waist circumference) and body composition evaluation (Fat Mass, Fat Free Mass, Total Body Water) were recorded during both visits. Body composition was measured through bioelectrical impedance analysis (BIA) device (BIA 101 Anniversary by Akern Srl, Firenze, Italy with the Bodygram 1.31 software and its equations by Akern Srl, Firenze, Italy).

### 2.2. Statistical Analysis

Descriptive statistics (i.e., mean and standard deviation, percentages of frequency) were used to summarize socio-demographic characteristics and anthropometric values.

Paired sample t-test was applied to evaluate the MD adherence score at T0 (“First visit”) and T1 (“Follow-up visit”). McNemar test was run to compare answer frequencies (%) to MD adherence questionnaire and FFQ at T0 and T1.

Chi-square analysis was used to assess the proportion in each group of the degrees of MD adherence and of adequate calcium intake, at T0 and T1. Adequacy of calcium intake was considered respect to Population Reference Intake (PRI) for the Italian population, reported by the Italian Society of Nutrition (SINU) in the Levels of Reference Consumption of nutrients and energy (Livelli di Assunzione di Riferimento di Nutrienti ed energia-LARN), (SINU, 2014) [12].

Statistical significance was considered *p* < 0.05. Analyses were performed using IBM SPSS Statistics for Windows, Version 20.0. (Armonk, NY, USA: IBM Corp.).

## 3. Results

Two hundred women were recruited. One hundred seventy-two (86%) were also evaluated at T1. The socio-demographic characteristics and lifestyle habits of the whole population at T0 and T1 are summarized in Table 1. The mean age at T0 was 61.60 ± 8.77 and at T1 was 61.72 ± 8.25.

At T0 evaluation, 93.5% of the women were in menopause, 17.5% had bone fragility fractures, and 73.5% supplemented with vitamin D. Moreover, mean weight was 60.9 ± 10.8 kg and mean BMI was 23.5 ± 3.8, which meant normal weight. Waist circumference was 92.1 ± 11.4 cm, Fat Mass was 26.7 ± 7.0%, Fat Free Mass was 72.9 ± 7.4% and Total Body Water was 53.7 ± 4.9%.

At T1 evaluation, mean weight was 60.3 ± 10.4 kg and mean BMI was 23.73 ± 3.9, which meant normal weight. Waist circumference was 91.24 ± 11.07 cm, Fat Mass was 27.2 ± 6.7%, Fat Free Mass was 72.8 ± 6.7% and Total Body Water was 53.3 ± 5.0%. Both results were comparable.

**Figure 2:**
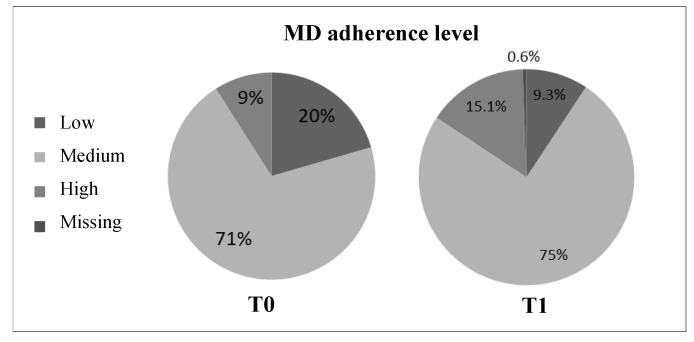
MD adherence level of peri- and post-menopausal women, at T0 (*N* = 200) and T1 (*N* = 171).

The MD adherence questionnaire showed that both at T0 and T1 a majority of the women had a medium MD adherence level (Figure 2). A chi-square analysis showed a significant statistical difference in the degrees of MD adherence both at T0 (χ_2_^2^ = 128.29, *p* < 0.001) and T1 (χ_2_^2^ = 137.39, *p* < 0.001). 

**Figure 3:**
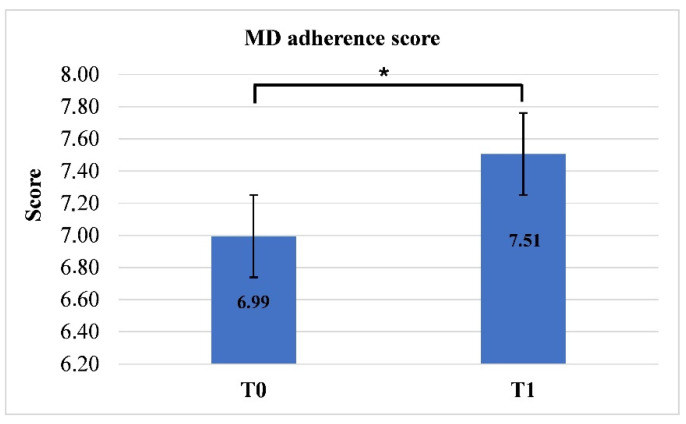
MD adherence score at T0 (*N* = 200) and T1 (*N* = 171) (Paired sample t-test; * *p* < 0.001).

Mean score of MD adherence at T0 and T1 was respectively 6.98 ± 1.74 and 7.53 ± 1.68. Paired sample t-test revealed a statistically significant difference between T0 and T1 (*p* < 0.001), so MD score increased (Figure 3). In particular, the consumption of vegetables and nuts significantly increased (respectively, *p* = 0.003 and *p* = 0.037), and daily consumption of red and processed meat decreased (*p* = 0.004). Also, the consumption of calcium rich mineral water increased significantly (*p* < 0.001).

The mean value of daily calcium intake was 866.2 ± 301.9 mg at T0 and 907.8 ± 304.8 mg at T1. At T0 and T1, participants were classified into adequate and inadequate groups (calcium intake higher than PRI for the Italian menopausal population or not) and then we assed statistical differences by means of chi-square analysis. The majority of participants had an inadequate calcium intake both at T0 (χ_2_^2^ = 102.24, *p* < 0.001) and T1 (χ_2_^2^ = 76.00, *p* < 0.001). 

**Figure 4:**
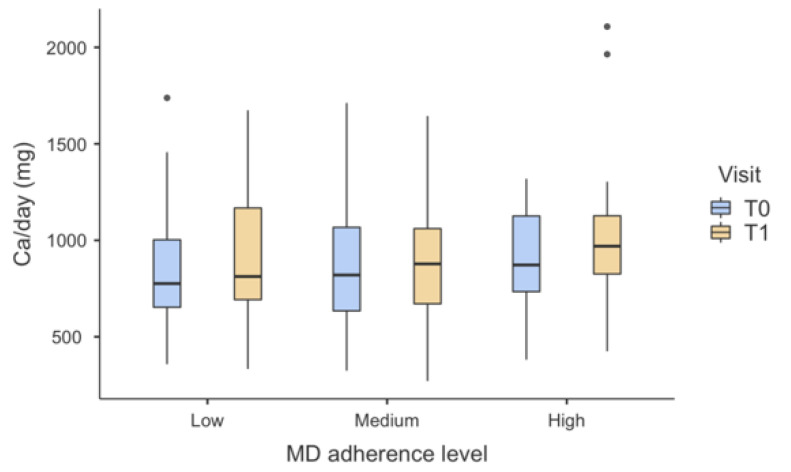
Box-plots chart showing dietary calcium intake in the three degrees of MD adherence, at T0 and T1. No statistically significant differences were observed. Outliers are represented by dots.

Figure 4 shows the dietary calcium intake as function of MD adherence level at T0 and T1 (it should be noted that taking into account other measures -such as demographic variables- the same trend was observed). No statistically significant differences were observed. Mean values (and standard deviations) at T0 were 839 ± 295, 870 ± 311, 900 ± 250 for low, medium, and high MD adherence, respectively. At T1, mean values (and standard deviations) were 914 ± 345 for low adherence, 885 ± 284 for medium adherence, and 1011 ± 368 for high adherence, showing an increase in calcium intake for high MD adherence from a descriptive point of view.

## 4. Discussion and Conclusions

The role of nutrition on bone health, especially the Mediterranean diet, has been widely discussed as it pertains to women during pre- and post-menopause [38,39]. Although a cause-effect relationship between MD and BMD and/or fragility fractures risk has not emerged, much evidence has proven the importance of a Mediterranean diet for bone health, encouraging the consumption of vegetables, fruits, nuts, and fish, rather than red and processed meats, sweet beverages, and high-energy density food [25,40].

Our study described the association between MD and dietary calcium intake in a population of two hundred peri- and post-menopausal women. Moreover, it highlighted the importance of preventive action (such as a 15–20 min nutritional conversation according to good clinical practice) to improve lifestyle habits and delay the consequences of osteoporosis.

This population showed a medium MD adherence level both at T0 (70.5%) and T1 (75%). The score increased significantly (6.98 ± 1.74 vs. 7.53 ± 1.68, *p* < 0.001), and could be suggestive of an amelioration of dietary habits. Dietary screening and nutritional intervention have often been useful for the management of chronic conditions, such as obesity and diabetes, and to improve lifestyle [41,42,43]. In this population, the consumption of healthy food groups also increased, such as vegetables (*p* = 0.003) and nuts (*p* = 0.037), and the consumption of red and processed meats decreased simultaneously (*p* = 0.004).

The majority of participants had an inadequate calcium intake both at T0 (866.2 ± 301.9 mg) and T1(907.8 ± 304.8 mg) respect to PRI for the Italian menopausal population (1200 mg per day). Our data are in line with Italian surveys on nutrients intake. To our knowledge, mean daily calcium intake in the Italian diet varies from 738 to 829 mg/day. The most recent Italian survey on food consumption was developed by INRAN (Istituto Nazionale di Ricerca per gli Alimenti e la Nutrizione-Italian Research Institute for Foods and Nutrition) during 2005–2006 [15,44,45]. In the adult population 18–65 years of age, calcium intake was about 800 mg/day for men and 730 mg/day for women, and 825 and 754 mg/day for men and women aged more than 65 years, respectively [15]. Other recent surveys reported data for specific disease groups, such as patients with inflammatory bowel disease [46], or adults with Type 1 Diabetes [47], or in regional populations. Castiglione et al. randomly collected data in a sample of 1838 subjects in the city of Catania, southern Italy, and reported that mean daily calcium intake among women was 798.23 mg, and 778.4 mg/for women aged more than 70 years [44].

It is interesting to highlight the consumption of calcium-rich mineral water increased significantly (*p* < 0.001) from T0 to T1. Although milk and dairy products give the greatest contribution, providing up to 80% of the total daily intake of calcium [44,45], natural mineral water with a calcium content ≥ 150 mg/L [48] may contribute to daily calcium requirements and has been demonstrated to be a good source of bioavailable calcium [49].

The positive contribution of the MD on bone health and osteoporosis prevention has been widely studied [24,40]. A recent systematic review on observational studies by Kunutsor et al. reported that limited evidence is available to demonstrate the beneficial effects of the MD on hip fractures [25]. Nevertheless, current literature has revealed that the consumption of some typical Mediterranean food groups (i.e., vegetables, fruits, nuts, low-fat dairy products, fish) is fundamental for the maintenance of a good bone health [27,28,29,30].

Our data show that, although not statistically significant, both at T0 and T1 a slight increase in calcium intake was observed for high adherence to MD diet from a descriptive point of view. Not only is the MD rich in plant origin foods, low-fat milk and dairy products, and fish, but the adherence to a healthier lifestyle may also sensitize populations to balanced dietary choices, which contribute to adequate daily nutrients intake [50].

This prospective study has some limitations: first, it is a correlational study, so a cause-effect relationship between MD adherence and calcium intake could not be established. A randomized controlled trial would be useful to deepen this theme. Moreover, in the study is not present a control group, that did not receive a nutrition conversation. This could have been useful to assess the effect of a short nutrition dialogue (15–20 min) on dietary habits change.

Finally, it should be noted that in our population a significant relation between MD and bone metabolism was not observed. Nevertheless, literature review and exploratory data suggest that MD could represent a sustainable and useful dietary pattern to ameliorate calcium intake. Further research is needed to prove hypothesized correlation and to acquire new data in order to apply it as a therapeutic adjunct to medication.

In conclusion, greater attention should be given to the diet as a complex of bioactive compounds and nutrients, and their interactive effects. In this context, the MD has emerged as the best choice to prevent many chronic diseases. An optimal calcium intake, according to the recommended daily allowance, and a dietary pattern in the Mediterranean style have proven their efficacy in preventing osteoporosis and maintaining good bone health.

The results in this population of peri- and post-menopausal women, show that a higher daily calcium intake is recorded in higher levels of MD adherence. In addition, a short duration (no more than 20 min) nutritional interview, during which advice was provided in the context of a good clinical practice routine, was sufficient to obtain promising results in terms of lifestyle improvement and prevention of osteoporosis.

## Figures and Tables

**Table 1:** Socio-demographic characteristics and lifestyle habits of the population at T0 and T1, descriptive statistics (percentages of frequency). No statistical differences between T0 and T1 were observed.

Variables	T0 (*N* = 200)	T1 (*N* = 172)
**Italian Nationality (%)**	98%	98.3%
**Marital status (%)**	Unmarried: 13.1% Married/cohabitant: 66.8% Separated/Divorced: 13.1% Widow: 7%	Unmarried: 14% Married/cohabitant: 66.1% Separated/Divorced: 12.9% Widow: 7%
**Educational status (%)**	Nothing: 1% Primary school: 13.6% Middle school degree: 21.6% High school degree: 47.7% Academic degree: 15.6% Post-academic degree: 0.5%	Nothing: 1.2% Primary school: 12.9% Middle school degree: 19.9% High school degree: 49.1% Academic degree: 16.4% Post-academic degree: 0.6%
**Smoking (%)**	12.1%	12.3%
**Physical activity Index (%)**	Inactive: 42.5% Moderately inactive: 16.5% Moderately active: 19.5% Active: 21.5%	Inactive: 48.2% Moderately inactive: 14.1% Moderately active: 15.9% Active: 21.8%

## Data Availability

The data presented in this study are available on request from the corresponding author.
